# Development and implementation of an end-of-shift clinical debriefing method for emergency departments during COVID-19

**DOI:** 10.1186/s41077-020-00150-0

**Published:** 2020-11-11

**Authors:** Jean-Christophe Servotte, T. Bram Welch-Horan, Paul Mullan, Justine Piazza, Alexandre Ghuysen, Demian Szyld

**Affiliations:** 1 Public Health Sciences Department, University of Liege, Liege, Belgium; 2 Interdisciplinary Medical Simulation Center of Liege, University of Liege, Liege, Belgium; 3 Director of Simulation, Section of Pediatric Emergency Medicine, Baylor College of Medicine, Texas Children’s Hospital, Houston, TX USA; 4 Director of Research and Quality Improvement, Division of Emergency Medicine, Children’s Hospital of the King’s Daughters, Eastern Virginia Medical School, Norfolk, VA USA; 5 Emergency Department, University Hospital Centre of Liege, Liege, Belgium; 6 Senior Director, Institute for Medical Simulation, Center for Medical Simulation, Brigham and Women’s Hospital, Harvard Medical School, Boston, MA USA

**Keywords:** Clinical event debriefing, Implementation, COVID-19, Communication, Safety, Quality

## Abstract

**Background:**

Multiple guidelines recommend debriefing after clinical events in the emergency department (ED) to improve performance, but their implementation has been limited. We aimed to start a clinical debriefing program to identify opportunities to address teamwork and patient safety during the COVID-19 pandemic.

**Methods:**

We reviewed existing literature on best-practice guidelines to answer key clinical debriefing program design questions. An end-of-shift huddle format for the debriefs allowed multiple cases of suspected or confirmed COVID-19 illness to be discussed in the same session, promoting situational awareness and team learning. A novel ED-based clinical debriefing tool was implemented and titled Debriefing In Situ COVID-19 to Encourage Reflection and Plus-Delta in Healthcare After Shifts End (DISCOVER-PHASE). A facilitator experienced in simulation debriefings would facilitate a short (10–25 min) discussion of the relevant cases by following a scripted series of stages for debriefing. Data on the number of debriefing opportunities, frequency of utilization of debriefing, debriefing location, and professional background of the facilitator were analyzed.

**Results:**

During the study period, the ED treated 3386 suspected or confirmed COVID-19 cases, with 11 deaths and 77 ICU admissions. Of the 187 debriefing opportunities in the first 8-week period, 163 (87.2%) were performed. Of the 24 debriefings not performed, 21 (87.5%) of these were during the four first weeks (21/24; 87.5%). Clinical debriefings had a median duration of 10 min (IQR 7–13). They were mostly facilitated by a nurse (85.9%) and mainly performed remotely (89.8%).

**Conclusion:**

Debriefing with DISCOVER-PHASE during the COVID-19 pandemic were performed often, were relatively brief, and were most often led remotely by a nurse facilitator. Future research should describe the clinical and organizational impact of this DISCOVER-PHASE.

## Background

In February and March of 2020, a global concern was that the COVID-19 pandemic could overwhelm available intensive care resources. To anticipate this challenge, many changes to standard operating procedures were required, and frequent, drastic workflow changes in the management of suspected or confirmed COVID-19 patients became the new paradigm [1]. Meanwhile, clinicians were exposed to contagion, high levels of stress and the psychological burden of managing both professional and personal duties [2, 3]. Such a volatile situation impacts healthcare professionals on the front line, especially those being exposed to potential COVID-19 patients in acute care environments such as the emergency department (ED) and inpatient settings [4].

Healthcare organizations and professionals aim to provide safe and efficient care [5]. Healthcare educators, clinicians, and leaders working at the intersections of education, quality improvement, and human factors are familiar with the role that effective non-technical skills have in preventing errors, enhancing patient safety, and improving resilience [6, 7].

Debriefing is a method to facilitate discussion of actions, guide reflection and transfer learning behaviors into clinical practice [8–10]. The American Heart Association (AHA) [11] and European Resuscitation Council (ERC) [12] recommend the use of debriefing to enhance clinical outcomes. These guidelines recommend debriefings in the minutes to hours after the clinical event [9]. Interdisciplinary debriefing after cardiac arrest events has been shown to improve patient survival [13]. Despite the growing recognition of these conversations as a good practice, debriefings occur infrequently [14, 15]. Cited obstacles to debriefing include a lack of time, a lack of trained facilitators, and a lack of debriefing locations [16, 17].

As part of the response to COVID-19, debriefing might help support resilience and teamwork, as well as contribute to improvements in quality and safety. Systematically incorporating team-based reflection, in the form of clinical event debriefing, into hospital workflows could address many of the patient safety and team adaptation challenges in the COVID-19 pandemic [18, 19]. This article aims to describe the development and implementation feasibility of a clinical debriefing system to inform ED leadership and support frontline teams. It details the international collaboration to develop the program and the local program implementation results, in two EDs in Liege, Belgium.

## Methods

The development of this clinical debriefing program began in January 2020. We (JC.S and DS) conducted a review of English-language literature using MEDLINE to identify clinical event debriefing concepts and principles, with the aim of developing and implementing a clinical debriefing tool specific to COVID-19. The search terms included “debriefing,” “clinical event,” “real event,” and “real-time.” A total of 205 articles were screened for inclusion. After titles and abstracts were reviewed, 16 articles were found to report concepts and principles related to clinical debriefing. Two researchers (JC.S and D.S.) reviewed the articles selected to aid in the design and implementation of a COVID-19-specific clinical debriefing tool. The conceptualization accelerated in mid-February 2020 when the likelihood of a pandemic was increasing. The researchers followed previously published recommendations for creating a clinical debriefing program in the ED [9].

### Why?

The goal of the debriefing program was to engage with frontline clinicians to identify latent safety threats, discover improvement opportunities, address systems-level interventions, and involve administrative support staff [15, 19–21]. Given that healthcare professionals reported increased levels of stress and anxiety during previous pandemics [22], this program could potentially create an opportunity to for team members to provide and receive peer support.

### When?

Determining the timing of when to gather team members to debrief can be challenging [9, 23]. Given the ED context, known for unpredictable acuity, shift work, and frequently changing team composition, we propose that instead of describing the timing of debriefings with an emotional temperature metaphor (*Hot, Warm,* or *Cold)*, clinical debriefing would better be described by the moment in time: Debriefing *After Shift Ends*. This option was selected because it represented a better balance between clinical workflows and bringing the whole team together as close in time as possible to clinical events (i.e., towards the end of a clinical shift), to allow time-sensitive information to be communicated. As such, the end-of-shift strategy was selected and qualified as clinical *Debriefing After Shift* Ends. Then, during the first wave of suspected patients, each end of shift was considered a debriefing opportunity.

### What?

To face the new clinical demands during the first wave, COVID-19 triage zones adjacent to the hospitals were created at the two sites on March 10. The debriefing program would convene frontline clinicians in the designated COVID zone and report key findings to ED leadership after each shift, creating a reflection opportunity for clinicians and a feedback loop for clinical leaders. Experienced healthcare simulation debriefers would facilitate short conversations (10–25 min) from their home or office. To record and identify the context of the shift, trained facilitators would document shift demographics and reactions of clinical team members. Team reflection was facilitated by encouraging episodes of self-reflection via the “plus/delta” analysis method [10]. This method was selected primarily because the facilitator was external to the clinical team and was not present on shift to observe the activity. Additionally, because the clinical team was composed of experienced clinicians, rather than novice learners, this method was likely to succeed at identifying valuable improvement opportunities [24]. The debriefing conversation was documented in real time to ensure accuracy. A one-page form was used to collect demographic data and serve as a cognitive aid to the facilitator by outlining the discussion structure and sample conversation scripts for the debriefing. As such, the model can be termed clinical debriefing *Plus-Delta in Healthcare After Shift Ends* (PHASE)*.*

### Who?

Based on the debriefing program objectives and previously reported best practices [9, 25], all team members actively involved in the management of COVID-19 patients in the ED should be invited to participate in the debriefing. In previous debriefing programs, a clinical team member, such as the physician team leader [10, 17, 26, 27] or the charge nurse [28] could serve as facilitator of the debriefing, but occasionally such a leader may prevent teammates from talking openly or may cause a reporting bias [9]. Given this concern and the local preferences of frontline clinicians, a debriefer with healthcare simulation experience from outside the clinical team serves in the role of facilitator for each debriefing. Simulation debriefing experts are trained to create an environment that encourages psychological safety and data confidentiality [29–31]. An additional advantage of such a facilitator is that he or she would not have any competing clinical priorities at the time of facilitating the debriefing. In this initial implementation, the facilitator would be one of the researchers (JC.S.), whose clinical background was in ED nursing and simulation. Before travel and work restrictions were implemented, the facilitator visited each COVID zone and was oriented to the medical equipment and PPE and the planned protocols. While external to the teams being debriefed, the facilitator had prior experience working with team members in the simulation setting.

### Where?

Options that were considered for where to debrief included the “dirty area” of the COVID zone, the “clean area,” or a meeting room outside of the COVID zone. While the first option, the dirty zone, was prohibited due to the risk of contamination, no decision was reached initially. Team members would be located together online or via teleconference in the ED to connect with the experienced simulation debriefer. Lifesize® (Lifesize, Inc., Austin, TX, USA) and/or Zoom® software (Zoom, Inc., San Jose, CA, USA) would be used for audio-video conferences. If these preferred methods failed, cellular phone calls or smartphone-based video conferencing (WhatsApp®, Inc., Menlo Park, CA, USA) would be used. In summary, the plan was to use *Debriefing In Situ* during *COVID-19 to Encourage Reflection* (DISCOVER).

### How?

In early March of 2020, it appeared that the pandemic would reach Europe. At that time, the conceptualization phase focused on how to create a standardized debriefing model. This model should be (1) simple to use for simulation educators; (2) clearly structured and brief, with an intended duration of 10–25 min; and (3) led by a debriefing facilitator who was ideally not on a clinical shift during the time of debriefing.

The literature review included some clinical debriefing tools that had been implemented elsewhere, some of which made clear use of a “plus/delta” analysis method. With efficiency of implementation in mind, the Debriefing In Situ Conversation after Emergent Resuscitation Now (DISCERN) tool [10] was selected. The researchers (JC.S and D.S) contacted two colleagues who had done research on the DISCERN tool and its subsequent adaptations [10, 15, 19, 21] (P.C.M. and T.B.W-H.) and invited them to join the research team. A teleconference was scheduled on March 1, 2020, to further define the project, clarify logistical aspects of the work, and develop on the standardized debriefing model.

After this initial meeting, a first draft of the debriefing model was designed (JC.S and D.S.). A period of remote work with iterative feedback through four videoconferences incorporated the DISCERN tool [10, 15, 19, 21] in the *Debriefing In Situ COVID-19 to Encourage Reflection and Plus-Delta in Healthcare After Shifts End* (DISCOVER-PHASE), developed the overall structure and the scripted language of the debriefing form.

On March 13, DISCOVER-PHASE was sent to three Belgian emergency physicians and simulation instructors, to a Belgian psychologist, and to experts from the Center for Medical Simulation (Boston, MA, USA) for review. On March 18, the Belgian government announced a stay-at-home order for the population because of COVID-19. That same day, the experts in Belgium and the USA approved the debriefing model.

#### Description of DISCOVER-PHASE clinical debriefing

**Fig. 1:**
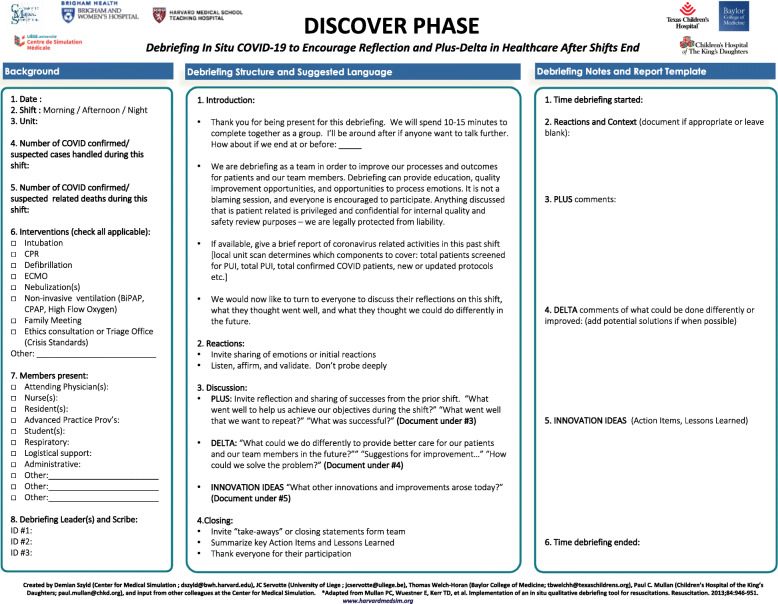
Debriefing In Situ COVID-19 to Encourage Reflection and Plus-Delta in Healthcare After Shifts End (DISCOVER-PHASE)

The DISCOVER-PHASE is a three-part structure (Fig. 1).

The first section concerning the "Background” collects demographic data, including the date and time of the debrief, team members present, debriefing facilitator name, and location of the clinical unit, as well as the number of COVID cases (confirmed and suspected) treated during the shift, any related deaths, and the clinical interventions performed by the team.

The second section is called “Debriefing Structure and Suggested Language” and has four stages: introduction, reactions, discussion, and closing. The Introduction aims to establish psychological safety and confidentiality as well as to share a few words appreciating the efforts of the team. During the reactions stage, the facilitator invites team members to share their emotions and initial reactions. In the case of a heightened reaction, the facilitator can offer to discuss the matter after the debriefing and/or provide referral resources. Subsequently, the facilitator asks team members to share the context of the shift they worked, including patient volume and acuity, as well as team composition. Next, the facilitator manages the discussion to balance participant talking, listening, reflecting, sharing, and learning. The conversation can focus on the pre-determined goals of the debriefing program, critical events, or topics from the reactions phase. The facilitator and team members can select from both “plus” comments (what went well) and “delta” comment (what could be done better next time) as topics by which to expand discussion further. Lastly, the facilitator closes the session by summarizing actionable suggestions, thanking everyone for their participation, and remaining available afterwards to anyone who wants to discuss any additional topics in a one-on-one format.

The third section of the form is titled “Debriefing Notes and Report Template.” The facilitator (or a colleague) documents the reactions and context during the discussion as appropriate. This section aims to provide a succinct and confidential account of “Plus-Delta” elements.

**Fig. 2:**
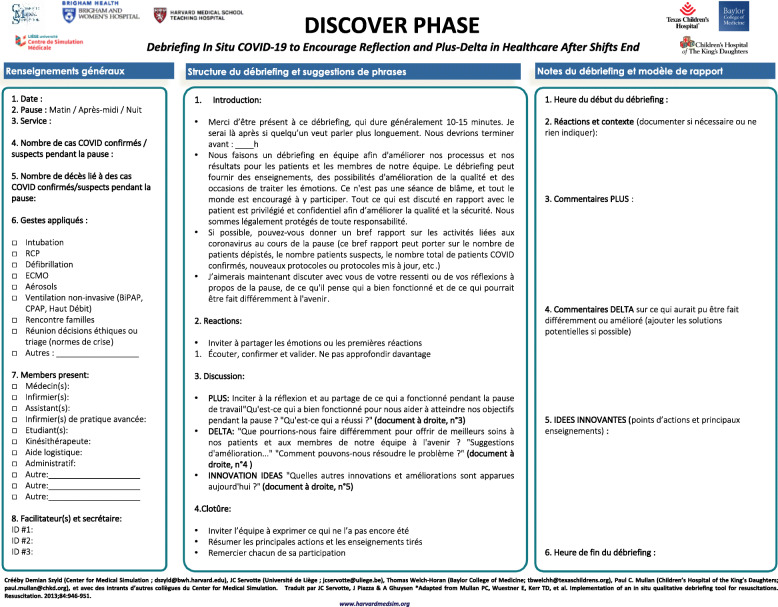
Debriefing In Situ COVID-19 to Encourage Reflection and Plus-Delta in Healthcare After Shifts End French Version (DISCOVER-PHASE-FR)

The text of DISCOVER-PHASE was simultaneously written (DS and JC.S) in English and French (Fig. 2).

#### Implementation of DISCOVER-PHASE in Belgium

The clinical debriefing program was first implemented in French in two EDs of the University Hospital of Liège (CHU), respectively named Sart-Tilman (CHU-ST; 622 inpatient beds) and Notre-Dame des Bruyères (CHU-NDB; 263 inpatient beds), with a combined annual ED census of 100,000 patients. CHU-ST is a tertiary care hospital located in the suburban area of Liege, while CHU-NDB is an urban secondary hospital. At both sites, a receptionist and two physicians were always present. There were four nurses scheduled in the daytime shifts and two during the overnight shift; two to three volunteer medical students came to reinforce the team throughout the day.

On the night of March 2, CHU-ST had its first patient with COVID-19. From March 2 to March 15, 594 suspected cases were cared for, with a gradual increase in cases over time. The first clinical debriefings were planned to start on March 19 in CHU-ST and on March 20 in CHU-NDB. The objective was to pilot test the DISCOVER-PHASE form as well as the audio-video conference system. A psychologist joined the first two debriefings in case their professional support was needed. Subsequently she remained available on an as-needed basis.

After each debriefing, the facilitator sent the report by email to the medical and nursing heads of the ED and the COVID-19 zones. He anonymized the comments and reminded stakeholders of the legal protections for the conversation according to local rules.

## Results

This article describes the initial results recorded during the first 8 weeks of the DISCOVER-PHASE program (March 16 to May 10). The results are summarized in Table 1. Among the 187 debriefing opportunities that occurred, a total of 163 (87.2%) debriefings were performed, evenly distributed between the two sites (93 at CHU-ST and 94 at CHU-NDB) (Table 1). Two additional debriefings were performed upon request (1.1%) in the early morning after night shift.

**Table 1:** DISCOVER-PHASE initial results

	Total	Week 1 (16–22 March)	Week 2 (23–29 March)	Week 3 (30 March–5 April)	Week 4 (6–12 April)	Week 5 (13–19 April)	Week 6 (20–26 April)	Week 7 (27 April–3 May)	Week 8 (4–10 May)
Number^a^	187	1 (0.5%)	23 (12.3%)	25 (13.9%)	27 (15.0%)	27 (13.4%)	29 (15.5%)	27 (14.4%)	28 (15.0%)
Shift^a^									
Morning (7 am–3 pm)	93 (49.7%)	1	12	11	14	13	15	13	14
Afternoon (3 pm–11 pm)	92 (49.2%)	0	11	12	13	14	14	14	14
Overnight (11 pm–7 am)	2 (1.1%)	0	0	2	0	0	0	0	0
Debriefing^a^									
Performed	163 (87.2%)	1	14	17	23	25	28	27	28
Not performed	24 (12.8%)	0	9	8	4	2	1	0	0
Unable to move to conference room	9	0	4	3	1	0	1	0	0
Clinical volume too high	8	0	4	3	1	0	0	0	0
Clinical volume too low	6	0	0	2	2	2	0	0	0
Debriefer not available	1	0	1	0	0	0	0	0	0
Cases handled	3386	477	713	592	353	285	312	332	322
Death	11	0	1	7	2	1	0	0	0
Intensive care unit	77	11	18	13	14	8	6	4	3
Audio-video conference system^a^									
WhatsApp	117 (62.9%)	0	5	16	17	13	22	20	24
Phone	37 (19.9%)	0	7	7	9	9	3	0	2
On site	19 (10.2%)	0	0	0	1	5	4	7	2
Lifesize	8 (4.3%)	0	6	2	0	0	0	0	0
Zoom	5 (2.7%)	1	4	0	0	0	0	0	0
Connection issue	1	0	1	0	0	0	0	0	0
Duration (min)^b^	10 (7–13)	20.0	21.5 (19.2–22.7)	13.0 (11.0–16.0)	10.0 (5.5–12.0)	8.0 (6.0–12.0)	9.0 (6.75–10.2)	10.0 (8.0–11.0)	10.0 (9.0–12.2)
Number of attendees^b^	5 (4–6)	4	5 (4–6)	4 (4–5)	5 (4–5)	5 (4–6)	4 (4–5)	5 (4–6)	5 (4–5)
Attendee roles^a^									
Nurse	163 (100%)								
ED resident	84 (51.5%)								
ED physician	64 (39.3%)								
Medical students	0 (0%)								
Other	0 (0%)								
Debriefer profession ^a^									
Nurse	140 (85.9%)	0	22	25	26	18	24	23	26
Physician	21 (12.9%)	0	0	0	1	9	5	4	2
Co-facilitation by nurse and psychologist	2 (1.2%)	1	1	0	0	0	0	0	0

^a^Frequency (%)

^b^Median (IQR)

**Fig. 3:**
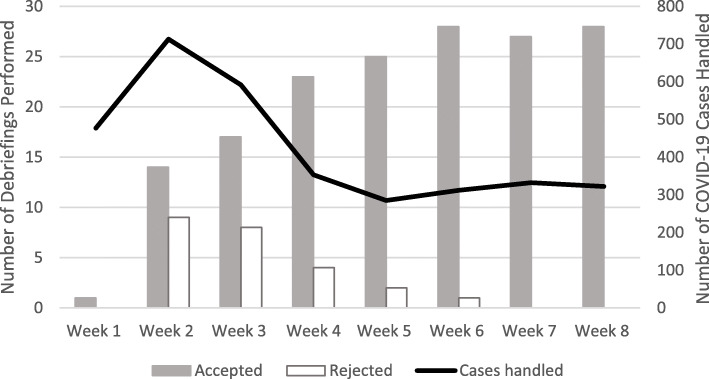
Number of COVID-19 cases handled and debriefings performed

The peak of COVID-19 activity in the ED of CHU occurred during week 2 (Fig. 3). This spike in activity corresponded to the gradual increase in debriefings proposed and actually performed. Usage of clinical debriefing continued to increase as the number of COVID cases decreased.

**Fig. 4:**
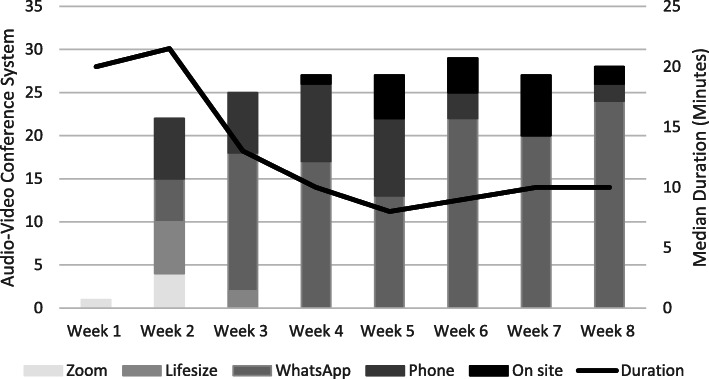
Debriefing median duration and audio-video conference system used

The largest numbers of debriefings occurred in weeks 6, 7, and 8. The clinical debriefings were predominantly facilitated by a nurse (85.9%), and starting in week 4, two emergency physicians also began leading clinical debriefings (21 out of 163; 12.9%). The median number of attendees was 5 (IQR, 4, 6) per debriefing and was stable over the eight weeks. In the 163 debriefings carried out, at least one nurse was always present (100%). Residents (51.5%) and supervisors (39.3%) also attended the debriefings frequently. Clinical debriefings had a median duration of 10 min. The durations were longer in the first 2 weeks and decreased by over 50% in the latter half of the study period. To perform the debriefing, Zoom® (2.7%) and Lifesize® (4.3%) were first used. In the middle of the second week, WhatsApp® Video Calls (62.9%) emerged as the most reliable communications method followed by cellular voice calls (19.9%). When physicians began to debrief, they were mainly on site (10.2%) (Fig. 4), rather than facilitating the discussion remotely.

Table 2 presents examples of situations described during the debriefings, debriefing facilitator actions and the responses from leadership. The facilitator informed stakeholders either for action or for awareness. With this information, leadership was able to respond accordingly for example creating educational material, revising protocols, and holding meetings.

**Table 2:** Examples of the “Delta’s” discussed during DISCOVER-PHASE debriefings and follow-up actions taken

Situation described in the debriefing	Debriefing facilitator actions	Response from leadership
Junior emergency medicine resident seen as unprepared to lead out-of-hospital assessment and treatment: - Perceived lack of skills in resuscitation and trauma management skills - Unaware or unfamiliar with standard protocols - Reliant on nurse for guidance	**Inform for action** - Telephone discussion with director of out-of-hospital emergencies to (resident lack of experience) **Inform for awareness** - Email to medical and nursing leadership	- Education (e-learning) created and distributed to the residents. - Protocol dissemination via email. - System revision (experienced nurse added to prehospital team and additional access for telephone backup).
Nurses, physicians, and residents noted problems with access to and understanding of current COVID-19 protocols - Information not readily available to clinicians - Updated protocols not applied in clinical setting	**Inform for action** - Email COVID-19 incident commander requesting information dissemination **Inform for awareness** - Email hospital leadership	- Implementation of daily briefing huddles at the beginning of each shift to explain the (new) protocols. - Creation of a password protected website that can be consulted remotely.
Critical patient treated in COVID-19 zone in standard room (not resuscitation room). Inefficient gathering of materials and equipment, unsatisfied team members (“felt chaotic”, “no clear leader outside medical team leader”)	**Inform for action** Described teamwork processes and learning opportunities - Lack of task allocation - Lack of identification of people and roles **Inform for awareness** - Call to the ED medical and nursing leaders to report what teams are experiencing	- Managers met with all physician leaders and encouraged them to explicitly plan for and communicate leadership tasks at the huddles at beginning of a shift and during resuscitations. - ED medical lead, with awareness of the problem and plan, met independently with physicians and supported manager’s request.
Transferring patients from COVID zone to radiology required coordination between ED nursing, radiology, and transport - Long waits for patients needed CT-scan (greater than 30 min) - Different standards applied by different disciplines (monitoring, PPE, hand-off)	**Inform for action** - Email to ED leadership suggesting review of ED-radiology interface and protocol **Inform for awareness** - An email detailing the issue was send to the ED medical and nursing leaders	- Meeting occurred between ED and radiology leadership with an agreement for radiology to call the ED at the time the CT scanner is available to avoid COVID patients in the hallway. - Nursing leadership provided education to nurses supporting the use of PPE for transport to avoid possible contamination of staff and patients.

## Discussion

This article reports on the development, format, implementation, and initial results of a clinical debriefing program after a shift ends: DISCOVER-PHASE. Of the circumstances that led to this work, three features are likely to persist during the COVID-19 pandemic and for the foreseeable future: uncertainty, changing clinical protocols, and social distancing. A method for clinicians to reflect on, identify and report quality and safety concerns that can be implemented remotely could enhance the ongoing response.

During the development of this debriefing program, our team addressed the various questions asked in a previously published guide for creating debriefing programs [9]. Its development and implementation were quickly carried out to meet the challenges caused by the initial peak of COVID-19. As we await a second peak of the pandemic, DISCOVER-PHASE is likely to remain central to the continued response for the institution at which it was first developed, and could support resiliency for providers and the health system. Healthcare managers elsewhere could implement debriefing programs in their own institution including via adapting this method based on their own local needs. Further research is needed to demonstrate the applicability and adaptability of our framework to other settings.

DISCOVER-PHASE is specifically designed to be used for end-of-shift clinical debriefing. The post-shift debriefing model is a routine or scheduled method, where the shift’s end serves as the “trigger to debrief,” rather than relying on patient-based “trigger” events that have formed the basis for previous debriefing tools (e.g., endotracheal intubation or cardiac arrest). This article is the first one we are aware of that defines the trigger for clinical debriefing based on the timing of the conversation. Moving to a routine approach could have contributed to the high acceptance rates we found by demystifying the activity and making it seem more routine and less intimidating to team members. The rate of accepted debriefings increased progressively over the study period as predicted by implementation science frameworks [32, 33]: adoption (week 1 and 2), implementation (weeks 3 to 5), and maintenance (after week 5).

A routine, end-of-shift debriefing strategy does not assume universal participation. Debriefings that were not performed should not be considered a negative outcome. We believe that the maintenance phase of our intervention was achieved fairly quickly for two reasons. The first is that there was commitment from debriefers and ED leadership to act on “deltas” (i.e., what could have gone better in the care of patients) and communicate solutions when possible. This is a new and innovative approach in the Belgian context, and it has required considerable effort on the part of the head of the ED. The second reason is the rapid adaptation of the debriefer during the adoption and implementation phases. ED team members were not previously accustomed to use the Zoom® and Lifesize® videoconferencing systems. Clinicians stated that they did not want to leave the COVID-19 zones to debrief. Driven by empathy, the debriefer literally met the clinicians where they were, by offering them the possibility of making calls via familiar methods such as WhatsApp® or by mobile phone. We believe that this practice contributed to enhance psychological safety and greater interest in program participation.

There are many different strategies for audiovisual communication ranging from elaborate, high-definition hardware to portable and inexpensive cellular technology. Debriefing remotely has been demonstrated successfully [34–36]. In addition to maintaining social distancing, minimizing exposure to infection, and decreasing the consumption of personal protective equipment, facilitating debriefings remotely for in situ clinicians allows a debriefer to work with units that are geographically distant. A facilitator wishing to debrief clinicians ending shifts early in the morning and late in the afternoon would need to have a very long day of work or commute twice. Working remotely provides the feasibility to debrief both day and night shifts.

We consider the high frequency of debriefings in our setting, despite the high case volume of the local COVID-19 peak (week 2), to be evidence of the feasibility and value of this program. We were intrigued to note that debriefings were sustained after the peak of COVID-19 cases when we expected a decrease in interest from our colleagues. The length of debriefings peaked early and declined rapidly, settling to a median of approximately 10 min by week 5, similar to debriefing durations found in previous studies [10, 37]. Over the 8-week period, clinicians may have increased their skills in reflection, as suggested by the predominance of accepted debriefings as well as the decrease in debrief length over time.

The clinical debriefings were facilitated by an external team member, rather than an attending physician or charge nurse present in the ED. The debriefers’ experience in healthcare simulation which values psychological safety and respects confidentiality may have contributed to the acceptance and success of the program. Moreover, as the simulation program was closed during this COVID-19 surge, our experience suggests an opportunity to redeploy educators virtually to the clinical setting during times of crisis.

During the first COVID-19 wave, clinical debriefings helped ED team members to reflect, to learn, and to feed forward actionable lessons to implement change. The facilitator used the “Debriefing Notes and Report Template” to inform the ED and hospital managers of proposals for improvement, innovations, etc. by sending it via an email clearly explaining to whom it was addressed [38]. Initially, we envisioned that the debriefing facilitator would send brief, templated email reports. During the course of the program implementation phase opportunities arose for the facilitator to meet with or call ED leadership to discuss more complex topics. Whether informing for action or awareness, at times a brief call seemed more appropriate than a lengthy email. We do not yet know how this practice impacts psychological safety or effectiveness of the program.

### Limitations

The clinical debriefing program was designed to identify quality improvement, teamwork, and educational opportunities during the COVID-19 pandemic, yet the current available data only detail local implementation. Further research is needed to investigate the impact on resilience, patient safety, and staff well-being. A multifaceted intervention such as this one, with a few critical design choices made in rapid fashion during a time of unprecedented change, needs further exploration using more multifaceted research outcomes. Moreover, in our initial work, most debriefings were conducted by the same person. It would be useful to explore whether the results can be replicated with other facilitators and in other contexts. This strategy relied on an external facilitator who was not part of the clinical team on duty but was aware of the protocols and well known to the staff and leadership as well as prior experience with debriefing. Facilitator characteristics may play a role to the implementation success of a clinical debriefing program.

## Conclusion

During the COVID-19 pandemic, we demonstrated the feasibility of rapidly implementing a routine, after post-shift clinical debriefing program in two emergency department locations. The novel practice of remote clinical debriefings was largely accepted in the clinical context where our study occurred. Future research is needed to better understand the impact of clinical event debriefing on clinical and educations outcomes for both team members and organizations.

## Data Availability

The dataset supporting the conclusions of this article is included within the article.
